# Assisted reproduction causes placental maldevelopment and dysfunction linked to reduced fetal weight in mice

**DOI:** 10.1038/srep10596

**Published:** 2015-06-18

**Authors:** Shuqiang Chen, Fang-zhen Sun, Xiuying Huang, Xiaohong Wang, Na Tang, Baoyi Zhu, Bo Li

**Affiliations:** 1 Department of Obstetrics and Gynecology, Tangdu Hospital, the Fourth Military Medical University, Xi’an 710038, China; 2 Laboratory of Molecular and Developmental Biology, Institute of Genetics and Developmental Biology, Chinese Academy of Sciences, Beijing 100080, People’s Republic of China; 3 Shaanxi Institute for Food and Drug Control, Xi’an 710038, People’s Republic of China

## Abstract

Compelling evidence indicates that stress in utero, as manifested by low birth weight (LBW), increases the risk of metabolic syndrome in adulthood. Singletons conceived by assisted reproductive technology (ART) display a significant increase in LBW risk and ART offspring have a different metabolic profile starting at birth. Here, used mouse as a model, we found that ART resulted in reduced fetal weight and placental overgrowth at embryonic day 18.5 (E18.5). The ART placentae exhibited histomorphological alterations with defects in placental layer segregation and glycogen cells migration at E18.5. Further, ART treatments resulted in downregulation of a majority of placental nutrient transporters and reduction in placental efficiency. Moreover, the ART placentae were associated with increased methylation levels at imprinting control regions of *H19*, *KvDMR1* and disrupted expression of a majority of imprinted genes important for placental development and function at E18.5. Our results from the mouse model show the first piece of evidence that ART treatment could affect fetal growth by disrupting placental development and function, suggests that perturbation of genomic imprinting resulted from embryo manipulation may contribute to these problems.

Epidemiological studies showing stress in utero, as manifested by low birth weight (LBW) is associated with chronic disease in adulthood. These associations have led to the “fetal programming of adult disease hypothesis” that nutrition and other influences during critical times of fetal life set the tissues and organs for life^1^,^2^,^3^. Singletons conceived by assisted reproductive technology (ART) have a 2.6-fold greater risk of LBW compared with those conceived naturally, and ART offspring have cardiovascular and metabolic risk factors even in childhood^4^,^5^,^6^. The causes of LBW are still unclear, as it is difficult to determine whether these problems are related to the ART procedure or to parental factors^7^,^8^. Over 5 million ART babies have been delivered worldwide since the breakthrough of *in vitro* fertilization (IVF) technology in 1978, and the demand for ART is continually increasing. Therefore, it is utmost importance to understand the possible causes of LBW, particularly those that may be associated with ART procedures, from the viewpoint of improving the technology and minimizing the health risks of babies conceived by ART.

The placenta plays a critical role in controlling maternal-fetal resource allocation and mediating programming of the fetus for future disease^9^,^10^. Studies have showed that variations in gross placental morphology at birth can predict a wide range of disorders in later life^10^,^11^. It has been observed that human pregnancies conceived by ART show an increased incidence of anomalous placentation^12^,^13^,^14^. Further, larger placentae and higher placental weight/birth weight ratios were found in ART pregnancies^15^. These placental abnormalities may contribute to abnormal fetal growth and thus are an important cause of LBW.

Imprinted genes are known to play an important role in placental development and regulating the placental transport capacity thereby controlling the supply of nutrients to fetus^16^,^17^. DNA methylation is an epigenetic modification in mammals that regulates the expression of imprinted genes from only one allele in a parent-of-origin-specific manner^18^. It is evident that ART procedures can disrupt DNA methylation of the imprinted genes in mice. Loss-of-imprinting caused by embryo manipulations was found in mid-gestation extraembryonic tissues in mice^19^,^20^,^21^. With respect to loss-of-imprinting, both maternally and paternally expressed imprinted genes in placentae derived from *in vitro* manipulated mouse embryos were modified at embryonic day 10.5 (E10.5)^22^. However, it is still not known whether perturbation in genomic imprinting induced by ART affects placentation and placental function during mid-to-late-gestation.

In this study, using the mouse model, we have tested the hypothesis that LBW associated with ART infants may be attributed to placental maldevelopment and dysfunction which result from perturbation of genomic imprinting induced by ART. Our experiments were designed to study the effects of ART on the subsequent development of conceptuses during mid-to-late-gestation. We provide evidence that suboptimal ART conditions can disrupt mammalian epigenetic reprogramming and result in perturbation of placental development and function, which may in turn contribute to reduced fetal weight in mice.

## Results

### Experimental groups

To determine the impact of ART procedures on fetal and placental development, mouse blastocyts were generated under three different experimental conditions: 1) In the IVC group, eggs were fertilized *in vivo*, collected at the one-cell stage and cultured *in vitro* in KSOM+AA to the blastocyst stage. 2) In the IVF group, eggs were fertilized *in vitro* and then *in vitro* cultured in KSOM+AA to the blastocyst stage. 3) In the *in vivo* group (control group), eggs were fertilized and developed *in vivo* to the blastocyst stage and then collected. Sixteen blastocysts were subsequently transferred to the uteri of each pseudopregnant female mouse; the placenta and fetus were collected at embryonic day 14.5 (E14.5) or E18.5 for analysis.

### IVF group had higher abortion rates than control and IVC groups

**Figure 1:**
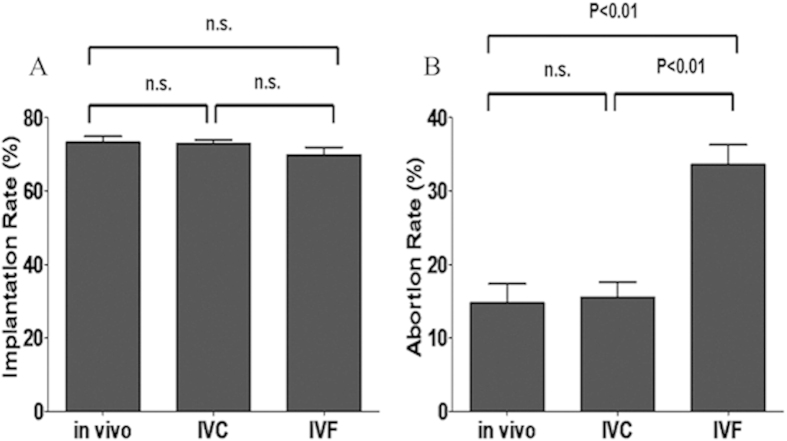
IVF resulted in higher abortion rate at E10.5. (**A**) Implantation rate of IVC, IVF and control groups at E10.5. (**B**) Abortion rate of IVC, IVF and control groups at E10.5. Data are presented as Means ± SD, groups with different superscripts differ significantly (p < 0.01).

Following transfer of the same number of blastocysts to the recipients, we compared the embryo implantation rates and the abortion rates after implantation between the IVC, IVF and control groups (*in vivo* group) at E10.5. We found that the embryo implantation rate was not different between the IVC, IVF and control groups (Fig. 1A). However, the fetal development rate after implantation was significantly lower in the IVF group than control and IVC groups (p < 0.01) (Table 1). The abortionrate of the IVF group was significantly higher than control and IVC groups (p < 0.01) (Fig. 1B). These results suggested that IVF can affect embryo survival and increase fetal loss after implantation.

### ART resulted in mouse placenta overgrowth and reduced fetus growth at E18.5

**Figure 2:**
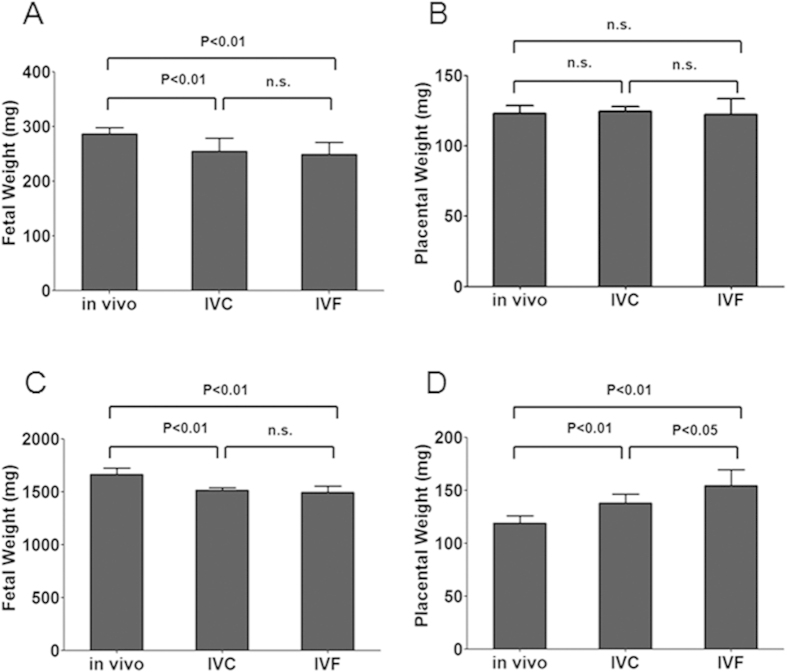
The impact of ART on fetal and placental development at E14.5 and E18.5. (**A**) E14.5 fetal weight and (**B**) E14.5 placental weight. The fetal weight was lower but the placental weight was no different in IVC and IVF groups at E14.5. (**C**) E18.5 fetal weight and (**D**) E18.5 placental weight. The fetal weight was lower at E18.5, but the placentae were significantly heavier in IVC and IVF mice at E18.5. Error bars show standard deviation (SD).

We analyzed the impacts of ART on placental and fetal development during mid-to-late gestation (Table 2). At E14.5, the fetal weight in the IVC and IVF groups was significantly lower than that in the control group (p < 0.01), but no significant difference was found between the IVC and IVF groups (Fig. 2A), and there were no differences between the groups with regard to placental weight (Fig. 2B). At E18.5, the fetal weight in both the IVC and IVF groups was significantly lower than that in the control group (p < 0.01), but no significant difference was found between the IVC and IVF groups (Fig. 2C). However, placentae from the two groups (IVC and IVF) were found to be significantly heavier than those from the control group (p < 0.01) (Fig. 2D). Moreover, at E18.5, the IVF placentae were significantly heavier than the IVC placentae (p < 0.05) (Fig. 2D), which suggests that IVF itself can contribute to abnormal placenta development. Thus, ART treatment can disturb mouse placental and fetal development at late gestation.

### ART leaded to abnormal placentation

**Figure 3:**
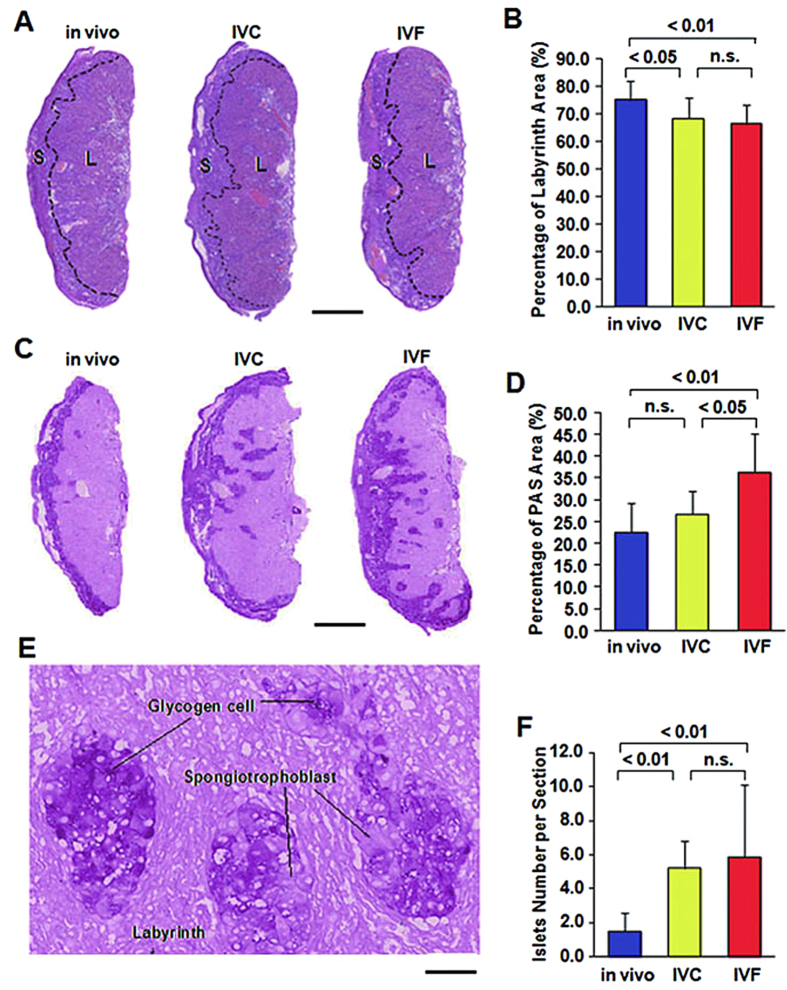
Morphologic analysis of E18.5 placentae. (**A**) HE staining of *in vivo*, IVC, and IVF placentae; S, Spongiotrophoblast layer; L, labyrinth layer (Scale bars: 500 μm). (**B**) The ratio of the labyrinth area/total area was reduced in IVC and IVF placentae. (**C**) Periodic acid Schiff (PAS) staining of *in vivo*, IVC, and IVF placentae. Glycogen cells are stained deep purple by PAS. Positive staining for PAS was observed in the decidua and junctional layer, and the IVF placentae exhibited a higher number of glycogen cell islets within the labyrinthine layer (Scale bars: 500 μm). (**D**) The ratio of the PAS-positive area/total area was increased in IVF placentae. (**E**) Magnified view of the islets, showing that the spongiotrophoblast layer islets were composed of two cell types, spongiotrophoblasts and glycogen cells (Scale bars: 50 μm). (**F**) The number of islets was increased in IVF placentae. Error bars show standard deviation (SD).

The mouse placenta consists of two fetal compartments (the labyrinthine zone and the spongiotrophoblast layer) and a maternal decidual component^23^. The spongiotrophoblast layer is composed of two major cell types, spongiotrophoblasts and glycogen cells. The glycogen cells accumulate glycogen within their cytoplasm from E12.5 and migrate into the maternal decidua during late gestation^24^. To determine whether ART affect placental morphology at late gestation, we performed H-E staining and Periodic acid-Schiff (PAS) staining to examine a large series of E18.5 placentae. In all the groups, the spongiotrophoblast layer and labyrinth zone were easily distinguishable (Fig. 3A). It was found that the IVF placentae groups had a significantly larger total area but a smaller labyrinth area/total area ratio compared with the placentae in the control group (0.66 ± 0.07 vs. 0.75 ± 0.07, respectively; P < 0.05; n = 10 and 10; Fig. 3B). There was significantly different between the IVC and control groups (0.68 ± 0.08 vs. 0.75 ± 0.07, respectively; P < 0.05; n = 10 and 10; Fig. 3B). Moreover, there was no significant difference between the IVC and IVF groups (P > 0.05). Additionally, the IVC and IVF placentae showed a distorted boundary between the spongiotrophoblast layer and labyrinth zone. We then used PAS staining to better define the defects in the spongiotrophoblast layer and labyrinth zone of the IVC and IVF placentae. The PAS-stained sections were particularly useful in visualizing the spongiotrophoblast layer because the layer stained magenta and a darker purple were compared to the labyrinth^25^,^26^. The spongiotrophoblast layer was positive for PAS, which suggests that many of these spongiotrophoblasts were glycogen cells (Fig. 3C). The ratio of glycogen-positive area /total area was significantly higher in the IVF (n = 10) placentae than the control (n = 9) and IVC (n  = 8) groups (0.344 ± 0.089 vs. 0.225 ± 0.068, P < 0.01; 0.344 ± 0.089 vs. 0.267 ± 0.052, P < 0.05; Fig. 3D). The ratio of glycogen-positive area /total area of the IVC group was higher than control group, but there was not significantly different between the two groups (0.225 ± 0.068 vs. 0.267 ± 0.052, P > 0.05; Fig. 3D). Islets of the spongiotrophoblast layer in the labyrinth zone were observed in normal placentae at E18.5. However, we noted that this feature was enhanced in the placentae of both the IVC and IVF groups (Fig. 3C). Moreover, IVC and IVF placentae exhibited numerous islets within the labyrinthine zone. These islets were composed of both glycogen cells (dark purple) and spongiotrophoblasts (light purple) (Fig. 3E). The number of independent islets was counted in every placenta of each group, and the results showed IVC and IVF placentae exhibited more spongiotrophoblast islets within the labyrinthine zone than the control group (5.2 ± 1.6 vs. 1.5 ± 1.1, P < 0.01; 5.9 ± 3.9 vs. 1.5±1.1 P  < 0.01) (Fig. 3F).

**Figure 4:**
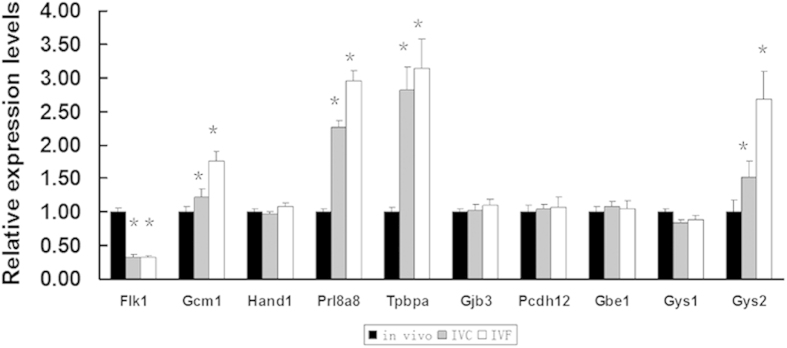
ART altered the relative expression levels of critical developmental genes in the placentae. The relative expression levels of critical developmental genes in the E18.5 placenta. *Flk1* marks the fetal vasculature and plays a key role in vascular development. *GCM1*, transcription factor which regulates placental cell fusion into the syncytiotrophoblast. *Hand1*, which is essential for giant trophoblast cell differentiation. *Prl8a8* and *Tpbpa*, which are expressed specifically in spongiotrophoblast cells. *Gjb3*/*Cx31* and *Pcdh12* are expressed exclusively in the glycogen cells of the mouse placenta. *Gys1*, *Gys2* and *Gbe1* are critical for glycogen metabolism. *p < 0.05. Error bars show standard deviation (SD).

To understand the molecular events responsible for the phenotypic alterations observed in placentae of the IVC and IVF groups, we analyzed and compared the expression level of placental marker genes in IVC and IVF placentae with that of the control placentae by quantitative RT-PCR (Fig. 4). The prolactin family 8 subfamily A member 8 (*Prl8a8*) gene, which is specifically expressed in spongiotrophoblast cells, was markedly up-regulated in both the IVC and IVF groups. Trophoblast-specific protein alpha (*Tpbpa*), a marker of the spongiotrophoblast layer that is expressed in both spongiotrophoblasts and glycogen cells, was also markedly up-regulated. These founds were consistent with the morphological alterations in the IVC and IVF placentae. The expression of the genes for several enzymes critical for glycogen metabolism, including glycogen synthases 1 and 2 (*Gys-1* and -*2*) and 1, 4--glucan branching enzyme (*Gbe1*), was comparable to the control group, and *Gys-2* was markedly up-regulated in placentae of the IVC and IVF groups, this may account for the increase of glycogen cells in ART-placentae. The kinase insert domain protein receptor (*Flk1*/*Kdr*), which is an indicator of fetal vasculature and plays a key role in vascular development, was significantly down-regulated in placentae of the IVC and IVF groups. The transcription factor glial cell missing 1 (*GCM1*), which is a marker for syncytiotrophoblasts in the labyrinthine zone, was significantly up-regulated. These results may reflect abnormalities in the labyrinthine zone in placentae of the IVC and IVF groups. The expression of heart and neural crest derivatives expressed transcript 1 (*Hand1*), which is essential for giant trophoblast cell differentiation, was not affected. Therefore, ART treatment can disrupt the development and structure of the mouse placenta.

### ART resulted in down-regulation of a majority of placental nutrient transporters and reduced the placental efficency

**Figure 5:**
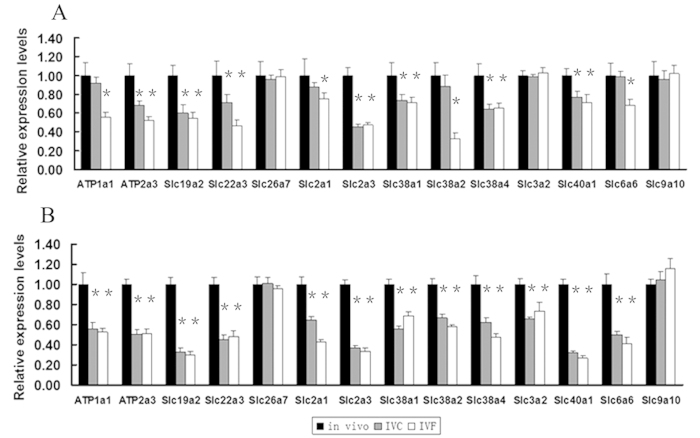
ART resulted in downregulation of most placental nutrient transporters and reduces placental efficiency. (**A**) The relative expression level of nutrient transporters in E14.5 placentae. *Slc38a1*, *Slc38a2*, *Slc38a4*, *Slc2a1*, *Slc2a3*, *ATP1a1*, *ATP2a3*, *Slc40a1*, *Slc19a2*, *Slc22a3* and *Slc6a6* expression was significantly decreased in IVF placentae, but the expression level of *Slc3a2*, *Slc26a7* and *Slc9a10* was not changed. (**B**) The relative expression level of nutrient transporters in E18.5 placentae. *Slc38a1*, *Slc38a2*, *Slc38a4*, *Slc2a1*, *Slc2a3*, *ATP1a1*, *ATP2a3*, *Slc40a1*, *Slc19a2*, *Slc22a3* and *Slc6a6* expression was significantly decreased in IVF placentae, but the expression level of *Slc26a7* and *Slc9a10* was not changed. *p < 0.05. Error bars show standard deviation.

The nutrient supply capacity of the placenta depends upon its size, morphology, blood flow and transporter abundance, all of which can vary naturally^27^. The activity of a range of nutrient transporters has been reported to be decreased in the placentae of fetuses with growth restriction^28^. Given that the ART-placentae had aberrant placental morphology, most notably in the labyrinth layer, and which is known to be crucial for the exchange of nutrients between maternal and fetal blood^29^, we tried to determine whether IVF affects the expression level of nutrient transporter genes. The expression of nutrient transporter genes in the placentae of the control, IVC, and IVF groups was compared at E14.5 and E18.5 (Fig. 5).

The expression of most nutrient transporter genes was significantly down-regulated in the placentae of both the IVC and IVF groups than the control group at E14.5 (Fig. 5A). In both IVC and IVF placentae, we found a significant decrease in the expression of solute carrier family 2 (facilitated glucose transporter) member 3 (*Slc2a3*/GLUT*3*), the System A family of amino acid transporters (*Slc38a1* and *Slc38a4*), Ca+*+* transporting ATPase (*ATP2a3*), iron-regulated transporter (*Slc40a1*), thiamine transporter 1 (*Slc19a2*), and organic cation transporter 3 (*Slc22a3*). Further, the expression level of Na(+)/K(+)-ATPase (*ATP1a1*), solute carrier family 2 (facilitated glucose transporter) member 1 anion exchange transporter (*Slc2a1*), system A amino acid transporter (*Slc38a2*), and sodium- and chloride-dependent taurine transporter (*Slc6a6*) was significantly down-regulated in the IVF group, but these genes had similar expression in the IVC and control groups. The expression of neutral amino acid transporter (*Slc3a2*), anion exchange transporter (*Slc26a7*) and sodium/hydrogen exchanger 10 (Slc*9a10*) was relatively unaffected.

In contrast, *Slc38a1*, *Slc38a2*, *Slc38a4*, *Slc2a1*, *Slc2a3*, *ATP1a1*, *ATP2a3*, *Slc40a1*, *Slc26a7*, *Slc9a10*, *Slc19a2*, *Slc22a3*, *Slc6a6* and *Slc3a2* were significantly down-regulated in the placentae in both the IVC and IVF groups at E18.5 (Fig. 5B). These results indicated that the affection of ART on the expression of nutrient transporter genes in the placenta were severer at E18.5 than E14.5.

**Figure 6:**
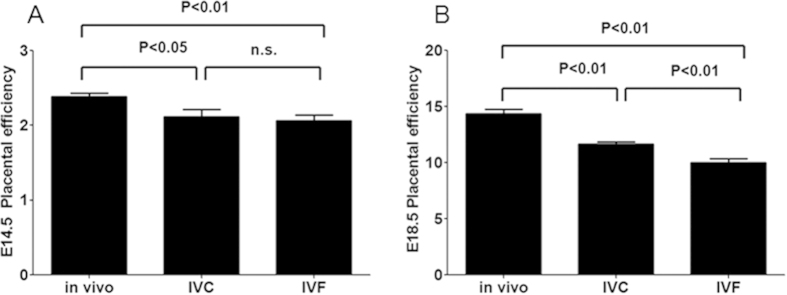
The impact of ART on placental efficiency. (**A**) Reduction in the placental efficiency of ART placentae at E14.5. (**B**) Reduction in the placental efficiency of ART placentae at E18.5.

In addition, placental efficiency (PE), calculated as “fetal/placental weight ratio” was used to measure nutrient transfer capacity from the placenta to the fetus^30^,^31^. We found significantly reduced PE in the IVC and IVF groups compared to the control group (p < 0.05) at E14.5 (Fig. 6A) and E18.5 (Fig. 6B). Moreover, the PE of the IVF group was significantly lower than that of the IVC group at E18.5 (p < 0.01) (Fig. 6B), which suggests that IVF itself could influence PE. In summary, our data show that ART can result in altered expression of placental nutrient transporters and reduce placental efficiency during mid-to-late gestation.

### ART increased DNA methylation level of ICRs and disrupted expression of imprinted genes in the placenta during mid-to-late gestation

Since imprinted genes are known to play a critical role in placental development and function^32^, we tried to determine whether ART leads to imprinting perturbation in mouse placentae at mid-to-late gestation. Here, we were interested in determining whether ART could lead to aberrant DNA methylation levels of imprinted genes in the placenta at mid-to-late gestation, as the effect of embryo manipulation on the methylation of *H19* ICR, *KvDMR1* and *SNRPN* ICR at mid-to-late gestation is still unclear. We analyzed the methylation levels of the *H19* ICR, *KvDMR1* and *SNRPN* ICR in placentae from the control, IVC and IVF groups at E14.5 and E18.5 by MassARRAY^®^. The MassARRAY^®^ application for quantitative DNA methylation analysis combines base-specific enzymatic cleavage with MALDI-TOF mass spectrometry^33^,^34^. The MassARRAY EpiTYPER application is scalable and allows to analyze multiple CpG sites on a single amplicon without compromising accuracy, sensitivity, or reproducibility^33^.

**Figure 7:**
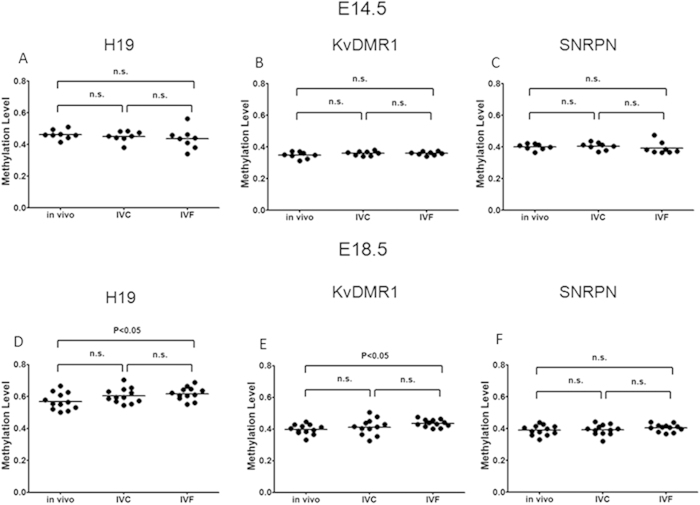
IVF altered DNA methylation at the *H19* ICR, *KvDMR1* and *SNRPN* ICR in E18.5 placentae. **(A)** Global methylation levels of the *H19* ICR were analyzed in E14.5 placentae from the *in vivo*, IVC and IVF groups. (**B**) Global methylation levels of *KvDMR1* were analyzed in E14.5 placentae from the *in vivo*, IVC and IVF groups. (**C**) Global methylation levels of the *SNRPN* ICR were analyzed in the E14.5 placentae from *in vivo*, IVC and IVF groups. (**D**) Global methylation levels of the *H19* ICR were analyzed in E18.5 placentae from the *in vivo*, IVC and IVF groups. (**E**) Global methylation levels of *KvDMR1* were analyzed in E18.5 placentae from the *in vivo*, IVC and IVF groups. (**F**) Global methylation levels of the *SNRPN* ICR were analyzed in E18.5 placentae from the *in vivo*, IVC and IVF groups.

The EpiTYPER assay returns quantitative methylation estimates of single CpGs or of small groups of adjacent CpGs (CpG units) depending on the sequence context. Using this method, we measured methylation levels of 13 CpG units, 32 CpG units and 11 CpG units in *H19* ICR, *KvDMR1* and *SNRPN* target regions respectively. We found that the mean methylation levels of CpG sites of the *H19* ICR, *KvDMR1*, and *SNRPN* ICR were not altered in the E14.5 placentae from the IVC and IVF groups compared with the control group (Fig. 7A,B,C). However, the mean methylation level of *H19* ICR and *KvDMR1* was significantly increased in the IVF placentae compared with the control group at E18.5. With regard to the 13 CpGs of the *H19* ICR, the average methylation level of the IVF placentae was significantly increased compared with the control placentae (0.62 ± 0.04 vs. 0.57 ± 0.05, respectively; n = 12 and 12; P < 0.05; Fig. 7D), but no significant difference was observed between the IVC and control groups (0.61 ± 0.05 vs. 0.57 ± 0.05, respectively; n = 12 and 12; P > 0.05; Fig. 7D). Similarly, no significant difference was observed between the IVC and IVF groups (P > 0.05). With regard to the 32 CpGs of the KvDMR1, the average methylation level of the IVF placentae was significantly increased compared with the control placentae (0.43 ± 0.02 vs. 0.40 ± 0.03, respectively; n = 12 and 12; P < 0.05; Fig. 7E), but no significant difference was observed between the IVC and control groups (0.41 ± 0.05 vs. 0.40 ± 0.03, respectively; n=12 and 12; P > 0.05; Fig. 7E). Similarly, no significant difference was observed between the IVC and IVF groups (P > 0.05). With regard to the average methylation level of the 11 CpG sites of the *SNRPN* ICR, no significant differences were found between the three groups (0.389 ± 0.031 in control group, 0.391 ± 0.033 in IVC group and 0.404 ± 0.023 in IVF group; n = 12, 12, 12 respectively; P > 0.05; Fig. 7F). Methylation levels of individual CpG sites within *H19* ICR, *KvDMR1* and *SNRPN* ICR at E14.5 and E18.5 were also evaluated. We also found that the methylation levels of some CpG sites were different between ART and control groups (Figure S1) and (Figure S2).

**Figure 8:**
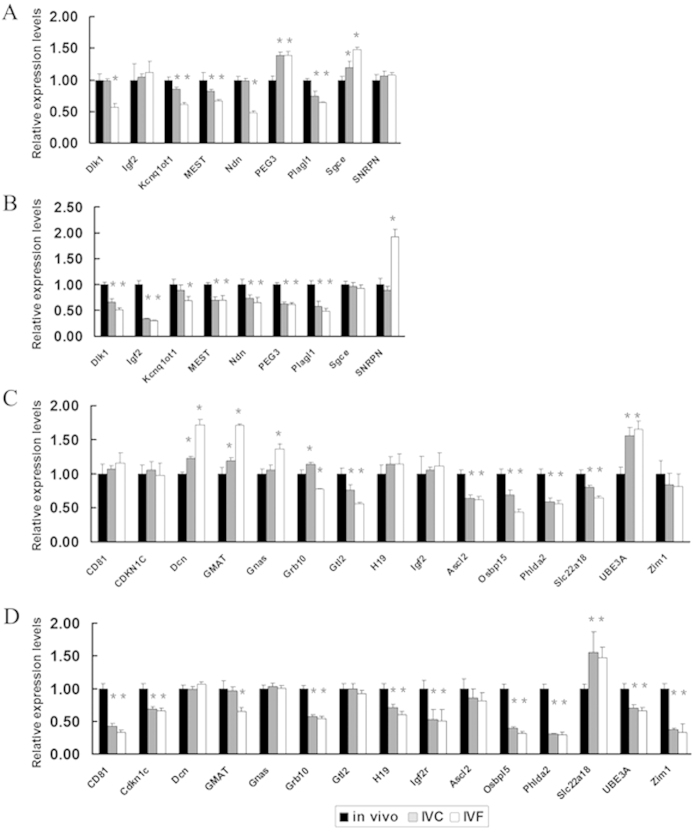
IVF placentae displayed significantly disturbed expression of imprinted genes. The expression levels of nine paternally expressed imprinted genes in E14.5 (**A**) and E18.5 (**B**) placentae and fifteen maternally expressed imprinted genes in E14.5 (**C**) and E18.5 (**D**) placentae from *in vivo* or *in vitro* fertilization and *in vitro* culture embryos. *p < 0.05. Error bars show standard deviation.

The expression of imprinted genes in the control, IVC and IVF groups was also compared at E14.5 and E18.5 (Fig. 8). At E14.5, the paternally expressed imprinted genes *PEG3* and *Sgce* were significantly up-regulated, whereas *Kcnq1ot1*, *MEST* and *Plagl1* were down-regulated in both the IVC and IVF groups compared with the control group. Interestingly, *Dlk1* and *Ndn* were down-regulated in the IVF group but unchanged in IVC group (Fig. 8A). Five of fifteen maternally expressed imprinted genes (*Gtl2, Mash2, Osbp15, Phlda2* and *Slc22a18*) were found to be significantly down-regulated in the IVC and IVF groups when compared with the control group. The expression levels of *CD81, CDKN1C, H19, Igf2r* and *Zim1* were mostly unchanged, whereas *Dcn, GMAT* and *UBE3A* were up-regulated in the IVC and IVF groups. *Gnas* was also up-regulated, but only in the IVF group. *Grb10* was up-regulated in the IVC group but down-regulated in the IVF group (Fig. 8C). At E18.5, six of nine paternally expressed imprinted genes (*Dlk1, Igf2, MEST, Ndn, PEG3* and *Plagl1*) were found to be significantly down-regulated in the IVC and IVF groups compared with the control group. *Kcnq1ot1* was also down-regulated, but only in the IVF group. The expression level of *Sgce* was mostly unchanged, whereas *SNRPN* was found to be up-regulated only in the IVF group (Fig. 8B). Nine of fifteen maternally expressed imprinted genes (*CD81, CDKN1C, Grb10, H19, Igf2, Osbp15, Phlda2, UBE3A* and *Zim1*) were found to be significantly down-regulated in the IVC and IVF groups compared with the control group. The expression level of *GMAT* was found to be down-regulated only in the IVF group, whereas the expression of *Dcn* and *Gnas* was mostly unchanged. The expression level of *Slc22a18* was up-regulated in the IVC and IVF groups (Fig. 8D). Our results therefore indicate that the placentae derived from ART embryos exhibit significant imprinting perturbations at mid-to-late gestation.

## Discussion

In this study, we show that ART is a significant factor responsible for abnormal placentation and function accompanied by genomic imprinting perturbation in the placenta at mid-to-late gestation in the mouse. Our data support the hypothesis that suboptimal ART treatment carries the risk of disruption in fetal development through abnormal development and function of the placenta.

In this study, to exclude the effect of fetus number on postimplantation development, we transferred eight embryos to each uterus in the control and experiment groups. After transfer to non-stimulated pseudopregnant mothers, fewer living fetuses were obtained from blastocysts in the IVF group. Moreover, the number of viable fetuses per female was lower in the IVF group, but the weight of IVF-derived fetuses was 13% lower at E14.5 (*P* < 0.01) and 10% lower at E18.5 (*P* < 0.01) in comparison with control fetuses. Our findings suggest that IVF treatment is an important factor that can contribute to reduced fetal weight.

In the present study, we have shown that ART treatment can lead to abnormal placentation and function. Firstly, we found that ART can lead to structural variations: IVC and IVF placentae had a significantly lower labyrinth/total area ratio, exhibited more spongiotrophoblast layers islets within the labyrinthine zone compared to the *in vivo* placentae. Especially, IVF placentae had significantly greater glycogen-positive area rate compared to the control placentae. To our knowledge, this is the first piece of evidence to show that ART treatment can cause increased placental glycogen content and result in structural abnormalities of the placenta. Secondly, the IVC and IVF placentae were significantly heavier than the control placentae at E18.5; however, no difference was noted at E14.5. Thirdly, the expression of a majority of nutrient transporters examined was significantly down-regulated in IVC and IVF placentae at E14.5 and E18.5. Further, the placental efficiency (fetal weight/placenta weight) was significantly decreased in the IVC and IVF groups at E14.5 and E18.5. This is in agreement with a report that amino acid and glucose transport was impaired in mouse IVF placentae^35^. We extended the finding by showing that not only amino acid and glucose transporter genes but also the transporter genes for the calcium transporter, iron transporter, thiamine transporter and taurine transporter were down-regulated in the IVC and IVF placentae. We thus conclude that ART treatment itself is a significant factor that can disturb mouse placental development and function.

Imprinted genes play a key role in determining placental phenotype and function, through regulate the differentiation and growth of many cell types within the placenta, particularly glycogen cells^34^,^36^,^40^. Imprinted genes also have direct effects on trans-placental transport via alteration of the expression of several different nutrient and ion transporters required for the facilitated and active transport of substances across the placenta^32^. Therefore, imprinted genes play a critical role in controlling maternal-fetal resource allocation and mediating programming of the fetus for future disease. In this study, we found that IVC and IVF disturbed the expression of a majority of imprinted genes in the placentae of normally developed E18.5 conceptus. Both maternally and paternally expressed imprinted genes were found to be modified and mostly down-regulated. The majority of down-regulated imprinted genes that we investigated are important for placental development and function, particularly for glycogen cell development, nutrients store and nutrients transport. Studies have showed that the paternally expressed *Dlk1*, which is a marker of sub-population of glycogen cells in placenta, is essential for nutrient metabolism^36^,^37^,^38^; *Igf2* is essential for glycogen cell development and has a key role in modulating the activity and expression of specific placental nutrient transporters^16^,^39^; *Cdkn1c*-mutant placentae have been reported to show an approximate doubling of spongiotrophoblast and labyrinthine cell number^40^; *Phlda2*-null mice exhibit placentomegaly with disproportionate expansion of the junctional zone and exhibit an abundance of stored glycogen^41^; *Phlda2* also regulates the expression of the glucose transporters *Slc2a1* and *Slc2a3* and is associated with LBW in humans^26^,^42^; Loss of expression of *H19* results in fetal overgrowth and placentomegaly with a 2.5-fold expansion of the glycogen cell population^43^. In this study, we found that *Dlk1*, *Igf2*, *Cdkn1c*, *Phlda2* and *H19* were significantly down-regulated in IVF placentae at E18.5, this may account for disproportionate expansion of the spongiotrophoblast layers and downregulation of nutrients transporters in the IVF placentae. Loss of the function of *MEST* results in placental weight deficit and late-gestation embryonic growth restriction, but the placental morphology otherwise appears normal^44^. We found that *MEST* was significantly down-regulated in IVF placentae at E14.5 and E18.5. Maternal inheritance of a disrupted *Grb10* (growth factor receptor-bound protein 10) allele results in placental and embryonic overgrowth^45^. In our study, down-regulation of *Grb10* was found in E18.5 IVF placentae. *Peg3*, *Plagl1* and *Meg3* are also important for placenta development and function^46^,^47^,^48^. We found that the expression of these three imprinted genes was perturbed in IVF placentae at E18.5.

Embryo manipulation will reduce the methylation levels of imprinted genes *H19* and *KvDMR1* in the preimplantation embryo and this impact upon placenta will last to 9.5 days of gestation (E9.5)^19^,^20^. It is believed that embryo manipulation will reduce the methylation levels of imprinted genes, resulting in an inherited “methylation pattern” that will be permanently incorporated into programmes of cell fate^49^,^50^. To our surprise, in this study, we found the E14.5 methylation levels of placental imprinted genes *H19* and *KvDMR1* in the IVF group were not significantly different from those of the control group, while the E18.5 methylation levels of placental imprinted genes *H19* and *KvDMR1* in the IVF group were significantly higher than those of the control group. These results indicated that methylation levels of the imprinted genes *H19* and *KvDMR1* in the IVF-origin placentae have been gradually increased from the mid-gestation to the late-gestation. Our study was the first to observe the dynamic process that placental methylation levels of the imprinted genes *H19* and *KvDMR1* in the IVF mice changed from being lower to being higher than those of the control group, during which process, compensatory placental overgrowth was accompanied. It is interesting that the increase of the methylation levels at these loci is linked to downregulation of the expression of *H19* and *Kcnq1ot1* and accompanied with placental overgrowth. Why and how the methylation dynamic change of imprinted genes was induced by ART deserve to be further study. This may be a common regulation mechanism with metabolism diseases of growth and development.

Abnormalities of ART placentae in humans have been described, including abnormal placental shape and abnormal umbilical cord insertion^15^,^51^,^52^. Larger placentae and a higher placental weight/birth weight ratio among pregnancies conceived by ART compared with spontaneous pregnancies also has been found in human^15^,^53^. The effect of ART on methylation and expression of imprinted genes in human placentae were also studied. An altered imprinted gene expression of *H19* and *PHLDA2* was observed in human placentae after IVF/ICSI, however, change in the methylation level of H19 was not found^54^. But another study has found that the methylation level of *H19* was reduced in human IVF/ICSI placentae^55^. These differences may account for sample size and different methods. Therefore, it is a common and conservative phenomenon that ART affects the placental functions in different species via epigenetic regulation and results in the abnormal fetal intrauterine growth. Thus, a further study that assesses ART’s impact upon placental development and function will give us a deeper understanding of the causes of ART-induced embryo-derived metabolic diseases and provide an entry point for the prevention of ART-induced embryo-derived metabolic diseases.

In summary, our work has demonstrated that ART manipulation can cause defects in placental layer segregation and glycogen cells migration at E18.5. These abnormalities in ART placenta are partly caused by perturbation of genomic imprinting resulted from embryo manipulation. Our work also demonstrated that dysfunction of the placenta is associated with lower fetal weight at E14.5 and E18.5, this is consistent with that singleton infants conceived through ART have a greater risk of LBW than those conceived spontaneously^4^. LBW is known to be linked to metabolic syndrome, such as hypertension, type 2 diabetes and obesity in adults. Many studies have reported an increased risk of metabolic disorders in IVF children and mice including a higher percentage of total body fat, higher systolic and diastolic blood pressure levels, and abnormal glucose metabolism^2^,^56^,^57^. Our work has demonstrated that abnormal placentation and function, partly caused by perturbation of genomic imprinting, may contribute to the LBW phenomenon associated with ART. Therefore, it is of utmost importance to elucidate the precise mechanisms as to how imprinting perturbations are induced by embryo manipulation and this would provide critical insights into ART-related developmental abnormalities and preventive actions.

## Materials and Methods

### Animals

Virgin 6- to 8-wk-old CD1 female mice, adult CD1 males, and Kunming vasectomized males were used. All animals were provided with nesting material and housed in cages maintained under a constant 12-h light/12-h dark cycle at 21–23 °C with free access to standard chow and tap water. The present study was approved by the Committee on the Ethics of Animal and Medicine of the Tangdu Hospital of The Fourth Military Medical University (Permit Number: TDLL-2013051), and was carried out in accordance with the Guidelines the Committee on the Use of Live Animals in Teaching and Research of the Tangdu Hospital of The Fourth Military Medical University. All efforts were made to minimize the number of animals used and their suffering throughout the course of the experiments.

### Experimental design

All female mice were superovulated and assigned to the IVF group, IVC group or control group. In the control group, blastocysts were collected after *in vivo* fertilization and *in vivo* development. In the IVF group, blastocysts were obtained after *in vitro* fertilization and development. In the IVC group, 1-cell embryos were harvested from the oviducts after *in vivo* fertilization and blastocysts were obtained after *in vitro* culture and development. All embryos were then transferred to pseudopregnant females.

### Superovulation

Females were superovulated by intraperitoneal injection of 7.5 IU (0.15 ml) Pregnant Mare Serum Gonadotropin (PMSG, ProSpec, Israel), followed by an intraperitoneal injection of 5 IU (0.1 ml) of human chorionic gonadotropin (hCG, ProSpec, Israel) 48 h later.

### *In vitro* fertilization

Conventional IVF was conducted using human tubal fluid (HTF) medium. The collected sperm from the cauda epididymis of adult male CD1 mice were suspended in HTF medium for at least 30 s and then placed in a dish in an incubator for capacitation. Capacitation of the spermatozoa was achieved by keeping them in a 37 ^o^C environment under 5% CO_2_ and 95% humidity for 1–2 h. The preincubated, capacitated sperm suspension was gently added to the freshly ovulated cumulus-oocyte complexes to obtain a final motile sperm concentration of 1–2 × 10^6^/ml, determined by a hemacytometer. Sperm and oocytes were co-cultured for 8 h in insemination medium.

### Embryo culture

*In vivo* or *in vitro* fertilized eggs (22–23 h after hCG), as determined by the presence of two pronuclei, were then cultured under optimized culture conditions (KSOM+AA, Millipore) and assessed for their developmental efficiency *in vitro*.

### Embryo transfer recipients

Kunming females of at least 6 weeks of age were mated with vasectomized Kunming males 4 days prior to embryo transfer. The morning after mating, females were checked for the presence of a vaginal plug, and on finding it, they were assumed to be on day 0.5 of pseudopregnancy. Embryos were then transferred to the uteri of the pseudopregnant females on pseudopregnancy day 3.5 according to standard procedures.

### Placenta dissection

Copulation was determined by the presence of a vaginal plug, and embryonic day zero (E0) was assumed to be midnight. Fetus and placentae were harvested at 2 different time points, E14.5 and E18.5. Pregnant mice were euthanized by CO_2_ inhalation followed by cervical dislocation. After dissection, placental and fetal wet weights were recorded, and each placenta was immediately preserved, some were fixed overnight in 4% paraformaldehyde/PBS for histological analyses, and the remainders were frozen in liquid nitrogen for mRNA and DNA detection.

### Histological and morphometric analyses of the placentae

Fixed placental discs were bisected through the attachment of the umbilical cord and embedded in paraffin wax. At least eight placentae (from different litters) in each group were examined. Five-micrometer cross-sections were obtained and stained with hematoxylin and eosin or periodic acid Schiff (PAS)-hematoxylin (both from Sigma-Aldrich). The stained sections were observed and photographed with a Leica microscope and camera (Leica SCN400; Leica Microsystems AG, Wetzlar, Germany). The border between the labyrinth and spongiotrophoblasts was identified visually and outlined using the Photoshop software (Adobe, San Jose, CA, USA). The total cross-sectional areas of the fetal and labyrinthine zones were measured and calculated using the ImageJ software (National Institutes of Health, Bethesda, MD).

### RNA extraction, cDNA preparation and real-time PCR analysis

A pool of six placental samples obtained from at least two litters from the same group was used total RNA extraction. RNA was extracted with Trizol (Invitrogen Life Technology) according to the manufacturer’s instructions and treated with DNase I to eliminate genomic DNA contamination. RT was accomplished using a commercially available first-strand cDNA synthesis kit (QIAGEN). The RT reactions were performed following the manufacturer’s protocol to reverse transcribe 1 μg of total RNA. Real-time PCR was performed with the Bio-Rad CFX96 real-time PCR instrument (Bio-Rad, Hercules, CA) and SYBR Green JumpStart Taq ReadyMix for Q-PCR according to the manufacturer’s protocol (Sigma-Aldrich). Gene expression was normalized using the housekeeping gene *Gapdh* as the reference gene. The primer sequences for the genes analyzed are listed in Table S1, Table S2 and Table S3. Primer sequences were obtained from Primer Bank or designed using Primer Express. Samples were analyzed using the ΔΔCt method.

### Methylation studies

Genomic DNA was extracted from placentae with the Blood and Tissue DNA Kit (QIAGEN, Dusseldorf, Germany) according to manufacturer’s instructions. The concentration and purity of the DNA were determined by absorbance at 260 and 280 nm. One microgram of DNA was bisulfite treated using the EpiTect Bisulfite Kit (Qiagen) following manufacturer’s protocol.

PCR primers were designed using the EpiDesigner software (Sequenom). The oligo sequences in this study

*H19* ICR (Forward: aggaagagagGTGTGATGTTTGTAATTATTTGGGAG, Reverse: cagtaatacgactcactatagggagaaggctTAAAATCCACAAACCCAACTAACCT), covering 13 CpG sites;

*SNRPN*ICR (Forward: aggaagagagTGGAGAGTTTTTTTGTTTAGTTTGG, Reverse: cagtaatacgactcactatagggagaaggctCACTCACTACCTTAATACTAACCACACC), covering 13 CpG sites;

KvDMR1 (Forward: aggaagagagTTGGAGTTTTTTTTAGGAATGTTGA, Reverse: cagtaatacgactcactatagggagaaggctCACAACCTCCTTCTCCAACTAAAAT), covering 32 CpG sites.

The amplified fragments were analyzed on a MassARRAY platform (Sequenom, San Diego, CA, USA) at the Beijing Genomics Institute at Shenzhen. Locus-specific PCR amplification was performed with the T7-promoters, where the latter was used to generate *in vitro* transcription on the amplified fragments. These transcripts were then subjected to enzymatic RNA base pair cleavage. The resulting fragments differ in size and mass depending on the sequence changes generated through bisulfite treatment. The fragment mass was determined by matrix-assisted laser desorption/ionization time-of-flight (MALDI-TOF) mass spectrometry.

The quantitative methylation data for each CpG site, or aggregates of multiple CpG sites, obtained were analyzed with the EpiTYPER software (Sequenom). The average methylation levels were obtained using weighted average.

### Statistic analysis

Quantitative data is expressed as Means ± Standard deviation (SD). The data were subjected to ANOVA test for assessing any significant difference among the three groups. The least significant difference (LSD) post hoc test was used to examine any significant difference between groups. Only probabilities lower than 0.05 were considered significant. The litter was served as the unit of comparison.

## Additional Information

**How to cite this article**: Chen, S. *et al*. Assisted reproduction causes placental maldevelopment and dysfunction linked to reduced fetal weight in mice. *Sci. Rep*. **5**, 10596; doi: 10.1038/srep10596 (2015).

## Supplementary Material

Supplementary Information

## Figures and Tables

**Table 1:** Implantation rates of the three groups.

Group	Litters	Stage	No. embryos transferred	Implanton rate (%)	No. of fetuses / No. of implantation (%)	No. of fetuses/No. of embryos transferred (%)	Abortion Rate (%)
**Vivo**	7	E10.5	112	73.3 ± 4.4 ^a^	85.2 ± 6.9 ^a^	62.5 ± 7.0 ^a^	14.8 ± 6.9 ^a^
**IVC**	7	E10.5	114	72.9 ± 2.8^a^	84.4 ± 5.4^a^	61.5 ± 3.2^a^	15.6 ± 5.4^a^
**IVF**	8	E10.5	119	69.8 ± 5.9 ^a^	66.5 ± 7.7^b^	46.9 ± 5.3^b^	33.6 ± 7.6^b^

Data are presented as Means ± SD, groups with different superscripts differ significantly (p < 0.01 by ANOVA).

**Table 2:** Litter size and fetal and placental weight.

Gestational age	Group	Litters	Litter size	Placental Weight (mg)	Fetal Weight (mg)
**E14.5**	Vivo	7	10.1 ± 1.3	123 ± 5^a^	287 ± 11^a^
	IVC	7	9.9 ± 1.4	124 ± 3^a^	255 ± 24^b^
	IVF	8	6.8 ± 1.3	122 ± 11^a^	250 ± 22^b^
**E18.5**	Vivo	6	10.5 ± 1.4	119 ± 7^a^	1665 ± 59^a^
	IVC	5	10.2 ± 1.5	138 ± 9^b^	1515 ± 25^b^
	IVF	8	7 ± 1.2	154 ± 15^c^	1491 ± 60^b^

Data are presented as Means ± SD, groups with different superscripts differ significantly (p < 0.05 by ANOVA).
