# Corrigendum: Assisted reproduction causes placental maldevelopment and dysfunction linked to reduced fetal weight in mice

**DOI:** 10.1038/srep29150

**Published:** 2016-07-20

**Authors:** Shuqiang Chen, Fang-zhen Sun, Xiuying Huang, Xiaohong Wang, Na Tang, Baoyi Zhu, Bo Li

Scientific Reports 5: Article number: 1059610.1038/srep10596; published online: 06182015; updated: 07202016

In the Supplementary Information file originally published with this Article, Tables S1–S3 were omitted. These errors have been corrected in the Supplementary Information that now accompanies the Article.

