# Supplementary material for: Assisted reproduction causes placental maldevelopment and dysfunction linked to reduced fetal weight in mice
Source: Sci Rep. 2015 Jun 18;5:10596. doi: 10.1038/srep10596 (PMC4471727; doi:10.1038/srep10596)
Supplement: Supplementary Information [file srep10596-s1.pdf]

Assisted reproduction causes placental maldevelopment and dysfunction linked to reduced fetal weight in mice

Shuqiang Chen[1,2,#], Fang-zhen Sun[2,#], Xiuying Huang[2], Xiaohong Wang[1], Na Tang[3], Baoyi Zhu[1], Bo Li [1,*]

1. Department of Obstetrics and Gynecology, Tangdu Hospital, the Fourth Military Medical University, Xi'an 710038, China

2. Laboratory of Molecular and Developmental Biology, Institute of Genetics and Developmental Biology, Chinese Academy of Sciences, Beijing 100080, People's Republic of China

3. Shaanxi Institute for Food and Drug Control, Xi'an 710038, People's Republic of China

#These two authors contributed equally to this work.

*Corresponding author

Bo Li, PhD

Department of Obstetrics and Gynecology,

Tangdu Hospital, the Fourth Military Medical University,

Xi'an 710038, China

Tel: +86-29-84777690

Fax: +86-29-84777690

E-mail: lbtn2000@126.com

Fang-zhen Sun, PhD

Laboratory of Molecular and Developmental Biology, Institute of Genetics and Developmental Biology, Chinese Academy ofSciences,

Beijing 100080, People's Republic of China

Tel: +86-10-64827885

Fax: +86-10-64827885

E-mail: fzsun_1962@126.com

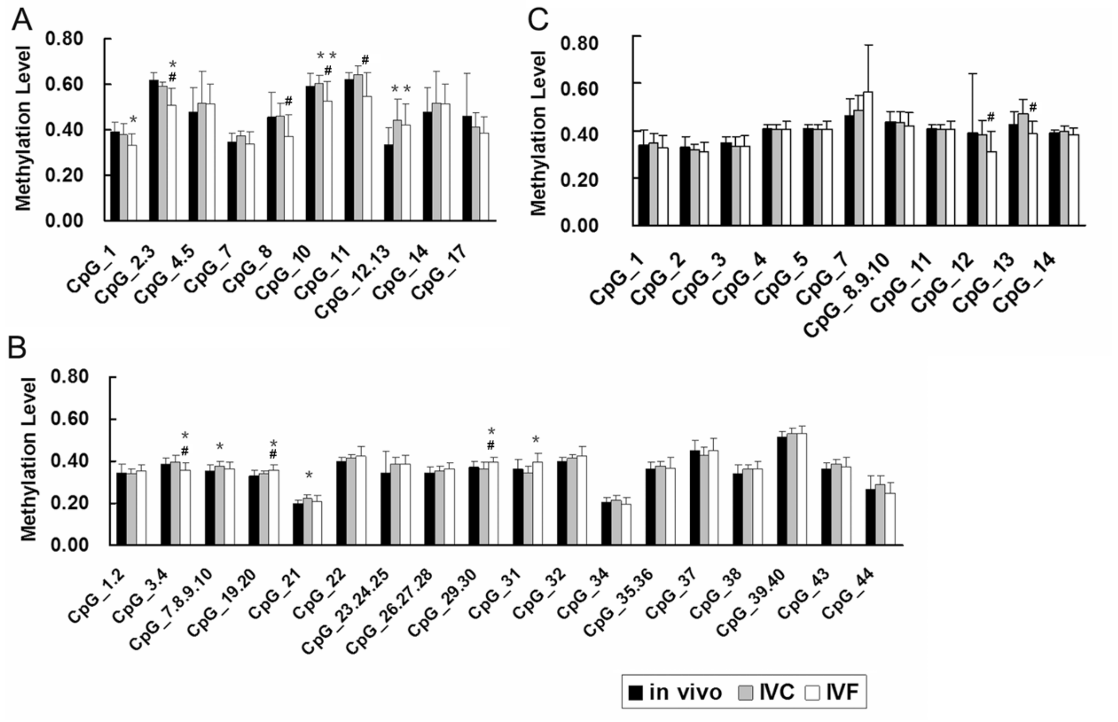

Figure S1. Methylation levels of individual CpG sites within H19 ICR, SNRPNIVR, and KvDMR1 at E14.5.

(A) Methylation levels of individual CpG sites in *H19* ICR were compared between samples from in vivo, IVC and IVF groups. **(B)** Methylation levels of individual CpG sites in KvDMR1 were compared between samples from in vivo, IVC and IVF groups. **(C)** Methylation levels of individual CpG sites in SNRPN ICR were compared between samples from in vivo, IVC and IVF groups. $^{*}p<0.05$ versus in vivo group; $^{\#}p<0.05$ versus IVC group. Error bars show standard deviation.

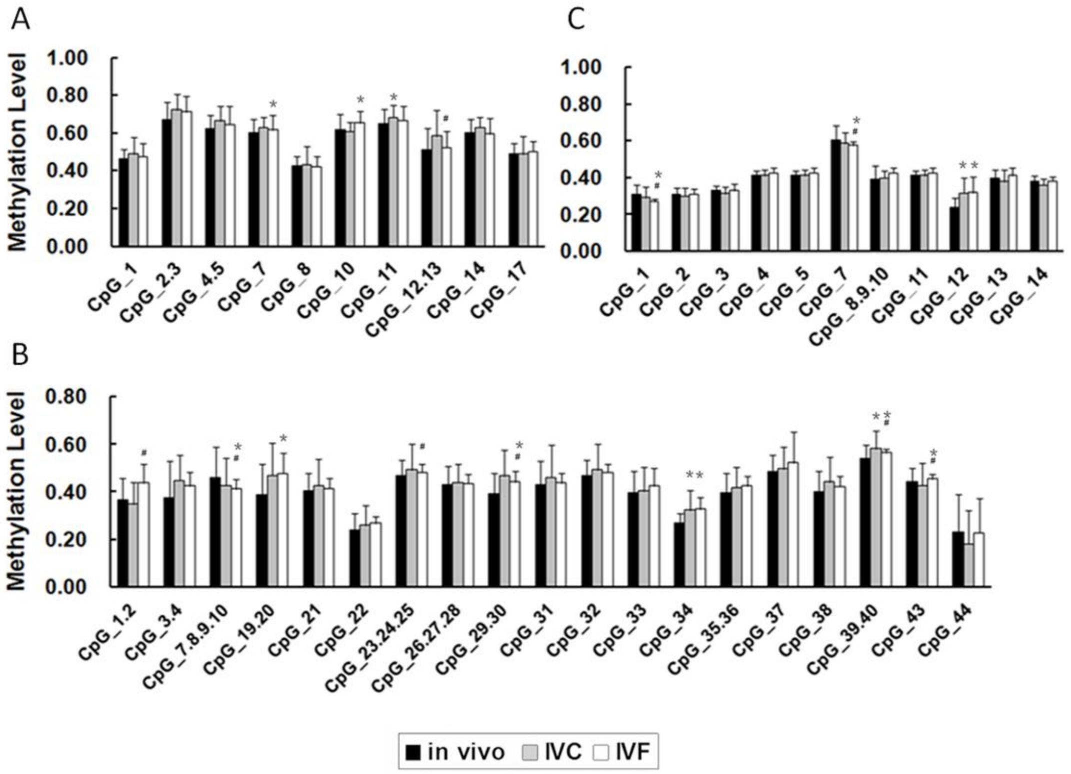

Figure S2. Methylation levels of individual CpG sites within H19 ICR, SNRPN ICR, and KvDMR1 at E18.5.

(A) Methylation levels of individual CpG sites in *H19* ICR were compared between samples from in vivo, IVC and IVF groups. **(B)** Methylation levels of individual CpG sites in KvDMR1 were compared between samples from in vivo, IVC and IVF groups. **(C)** Methylation levels of individual CpG sites in SNRPN ICR were compared between samples from in vivo, IVC and IVF groups. $^{*}p<0.05$ versus in vivo group; $^{\#}p<0.05$ versus IVC group. Error bars show standard deviation.
